# Supplementary material for: Myosin 2 drives actin contractility in fast-crawling species outside of the amorphean lineage
Source: bioRxiv. 2025 May 19:2025.05.16.654244. Preprint. [Version 1] doi: 10.1101/2025.05.16.654244 (PMC12139777; doi:10.1101/2025.05.16.654244)
Supplement: 3 [file NIHPP2025.05.16.654244v1-supplement-3.pdf]

bioRxiv preprint doi: https://doi.org/10.1101/2025.05.16.654244; this version posted May 19, 2025. The copyright holder for this preprint (which was not certified by peer review) is the author/funder, who has granted bioRxiv a license to display the preprint in perpetuity. It is made available under aCC-BY 4.0 International license.

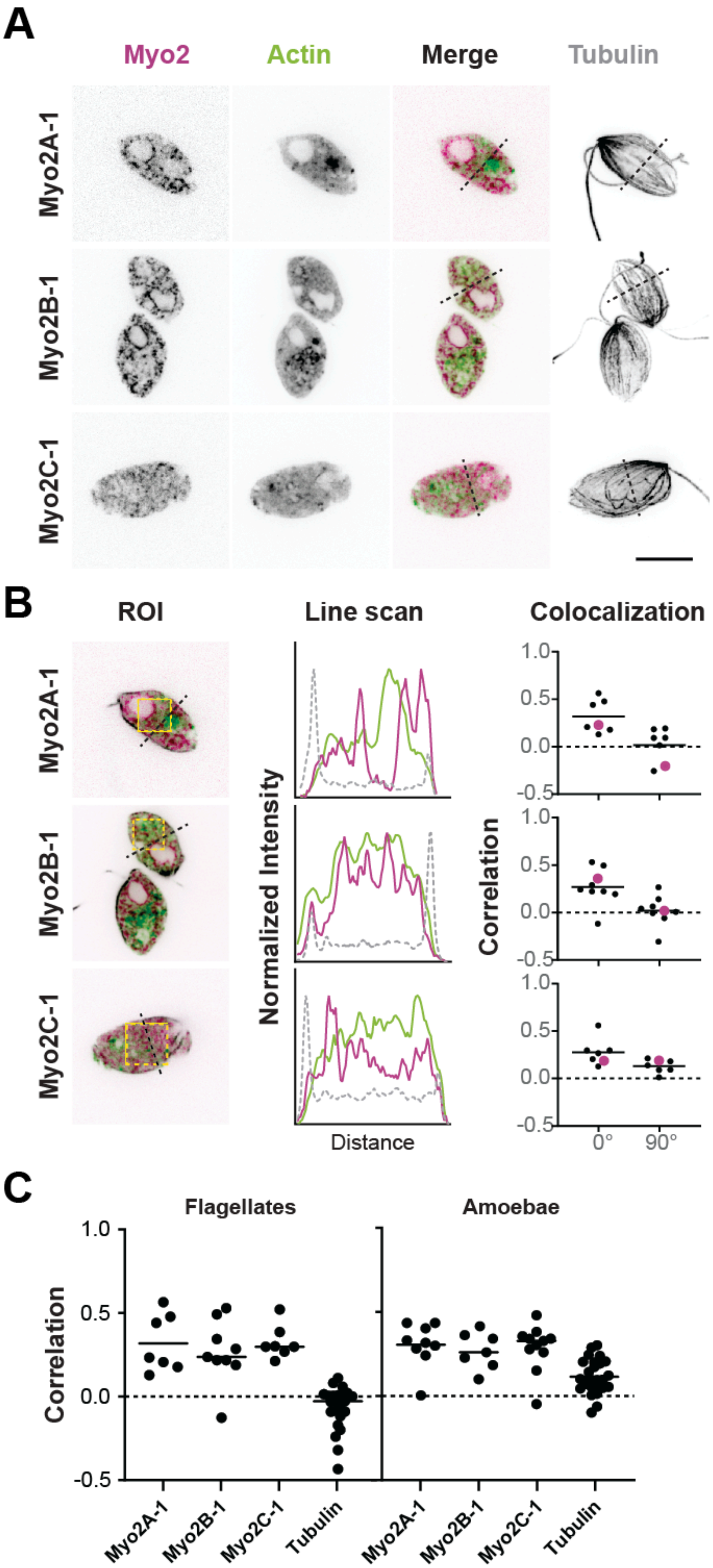

bioRxiv preprint doi: https://doi.org/10.1101/2025.05.16.654244; this version posted May 19, 2025. The copyright holder for this preprint (which was not certified by peer review) is the author/funder, who has granted bioRxiv a license to display the preprint in perpetuity. It is made available under aCC-BY 4.0 International license.

Figure S1: Myosin 2 localizes with actin in *Naegleria* flagellates. (**A**) *Naegleria* flagellates were fixed and stained with Myo2 antibodies (pink) to detect Myosin 2, DM1A to detect α-tubulin (gray), and Alexa Fluor™ 488 Phalloidin to label actin filaments (green). Single z slices through the midsection of cells are shown. (**B**) The Region of Interest (ROI) for each example depicts the area used for colocalization analyses; dotted yellow squares were used in 90° rotation tests, and dotted lines through the cell were used for line scans. The colocalization values are plotted as Pearson's correlation coefficients, with the representative cells shown on the left indicated by pink circles. The Myo2 and actin line scan fluorescence intensity was normalized to the dimmest (0%) and brightest (100%) signal for regions within the cortex boundary. ($n \geq 6$ cells for each antibody; scale bar = 10 μm). (**C**) A comparison of the Pearson's correlation coefficients for Myosin 2:actin between flagellates and amoebae shows that the Myosin 2 signal localizes weakly (<0.5) to actin in both cell types, using tubulin:actin localization as a negative control.

bioRxiv preprint doi: https://doi.org/10.1101/2025.05.16.654244; this version posted May 19, 2025. The copyright holder for this preprint (which was not certified by peer review) is the author/funder, who has granted bioRxiv a license to display the preprint in perpetuity. It is made available under aCC-BY 4.0 International license.

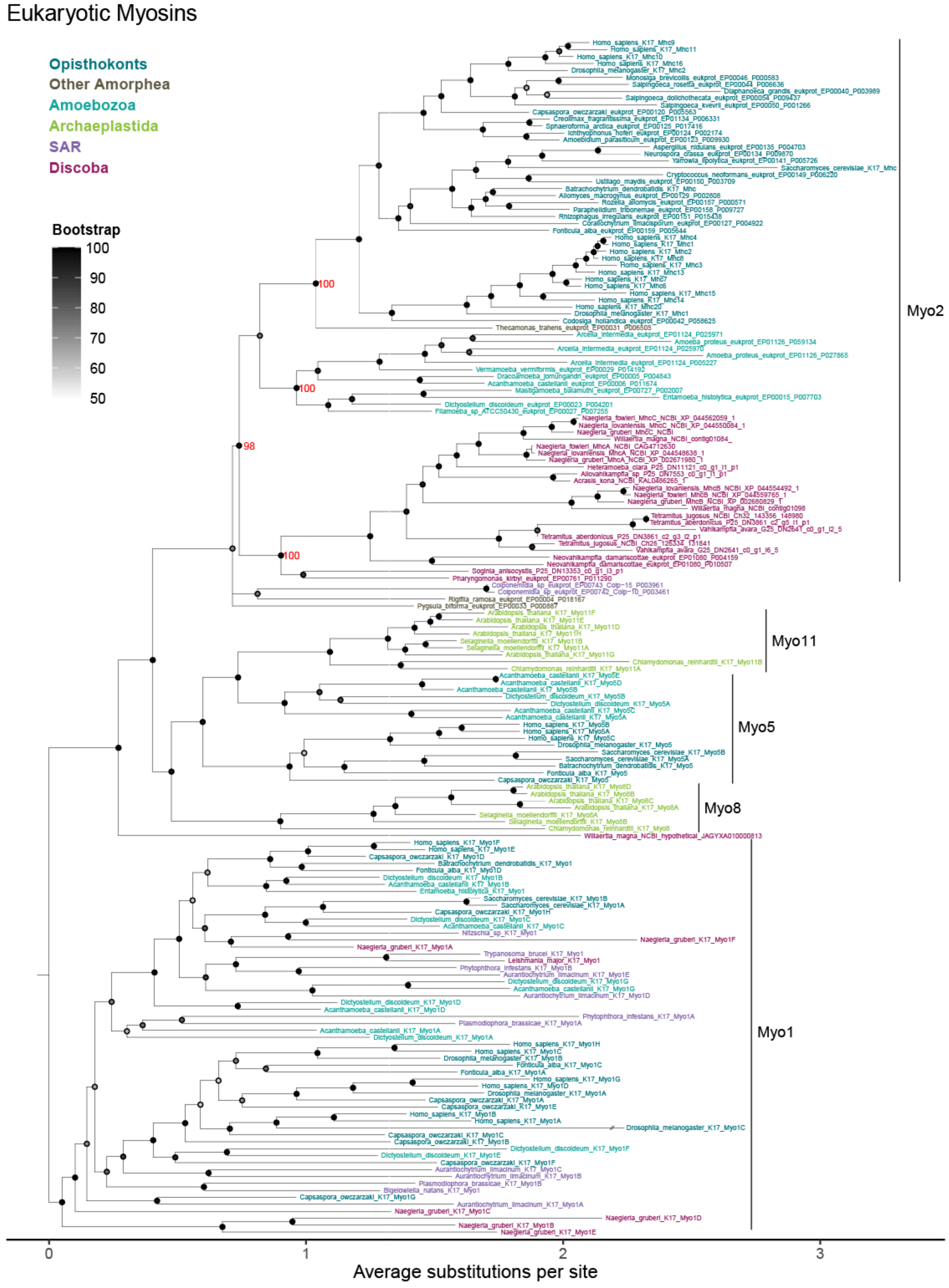

bioRxiv preprint doi: https://doi.org/10.1101/2025.05.16.654244; this version posted May 19, 2025. The copyright holder for this preprint (which was not certified by peer review) is the author/funder, who has granted bioRxiv a license to display the preprint in perpetuity. It is made available under aCC-BY 4.0 International license.

Figure S2: Heterolobosea encode direct orthologs to Amorphean Myosin 2s. Eukaryotic myosins were identified using an HMM built from Amorphean Myosin 2 sequences and are presented in a gene tree alongside other myosin types. The tree is rooted at the edge connecting the Myosin I branch to all other branches. Gene labels are color-coded by eukaryotic supergroup (upper left). Nodes with bootstrap support below 50% are collapsed into polytomies, and bootstrap values for key nodes are displayed in red. *Naegleria* and other heterolobosean Myosin 2s cluster with all other Myosin 2s (98% bootstrap support).

bioRxiv preprint doi: https://doi.org/10.1101/2025.05.16.654244; this version posted May 19, 2025. The copyright holder for this preprint (which was not certified by peer review) is the author/funder, who has granted bioRxiv a license to display the preprint in perpetuity. It is made available under aCC-BY 4.0 International license.

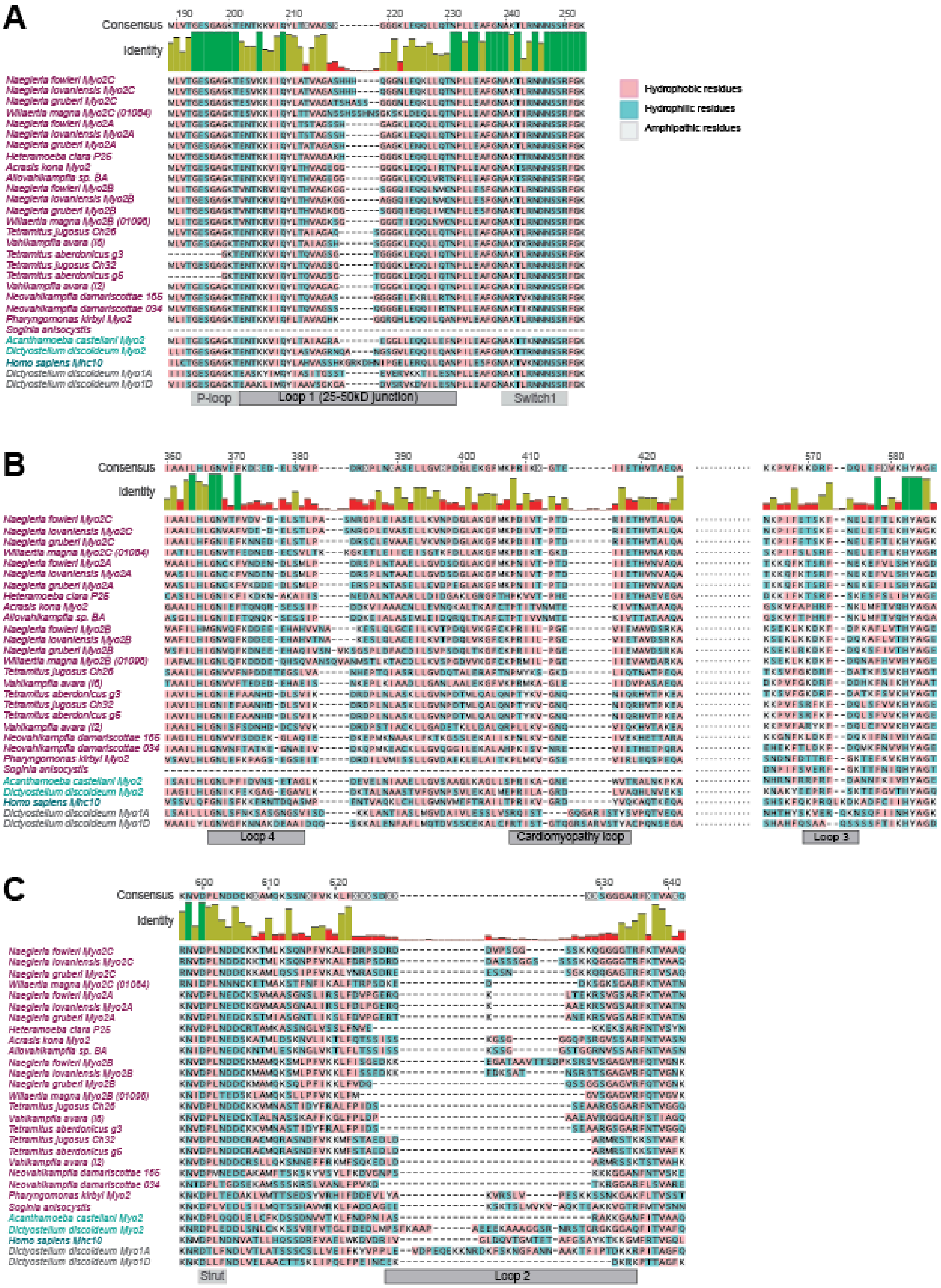

bioRxiv preprint doi: https://doi.org/10.1101/2025.05.16.654244; this version posted May 19, 2025. The copyright holder for this preprint (which was not certified by peer review) is the author/funder, who has granted bioRxiv a license to display the preprint in perpetuity. It is made available under aCC-BY 4.0 International license.

Figure S3: Heterolobosean Myosin 2 sequences vary from Amorphean Myosin 2s in their actin-binding regions. Myosin 2's actin binding is dominated by strong hydrophobic interactions between surface residues on actin and corresponding residues of the motor domain's actin-binding interface, including the 'cardiomyopathy (CM) loop' and loops 1- 4.[55,56] (**A-C**) A multi-sequence alignment using ClustalO shows heterolobosean sequence similarity of actin-binding regions: (**A**) Loop 1, (**B**) Loop 4, the cardiomyopathy (CM) loop, Loop 3 and (**C**) Loop 2. The residues are color-coded by hydrophobicity, with hydrophobic residues in pink, hydrophilic in blue, and amphipathic in gray. By pairwise comparison to *Dictyostelium*, the average heterolobosean sequence similarity was 70.2% (vs *Acanthamoeba* 75.8%) in Loop 1, 52.3% (vs *Acanthamoeba* 42.9%) in Loop 4, 51.9% (vs *Acanthamoeba* 63.6%) in CM loop, 59.4% (vs *Acanthamoeba* 50%) in Loop 3, and 28.0% (vs *Acanthamoeba* 44.4%) in Loop 2.

bioRxiv preprint doi: https://doi.org/10.1101/2025.05.16.654244; this version posted May 19, 2025. The copyright holder for this preprint (which was not certified by peer review) is the author/funder, who has granted bioRxiv a license to display the preprint in perpetuity. It is made available under aCC-BY 4.0 International license.

A

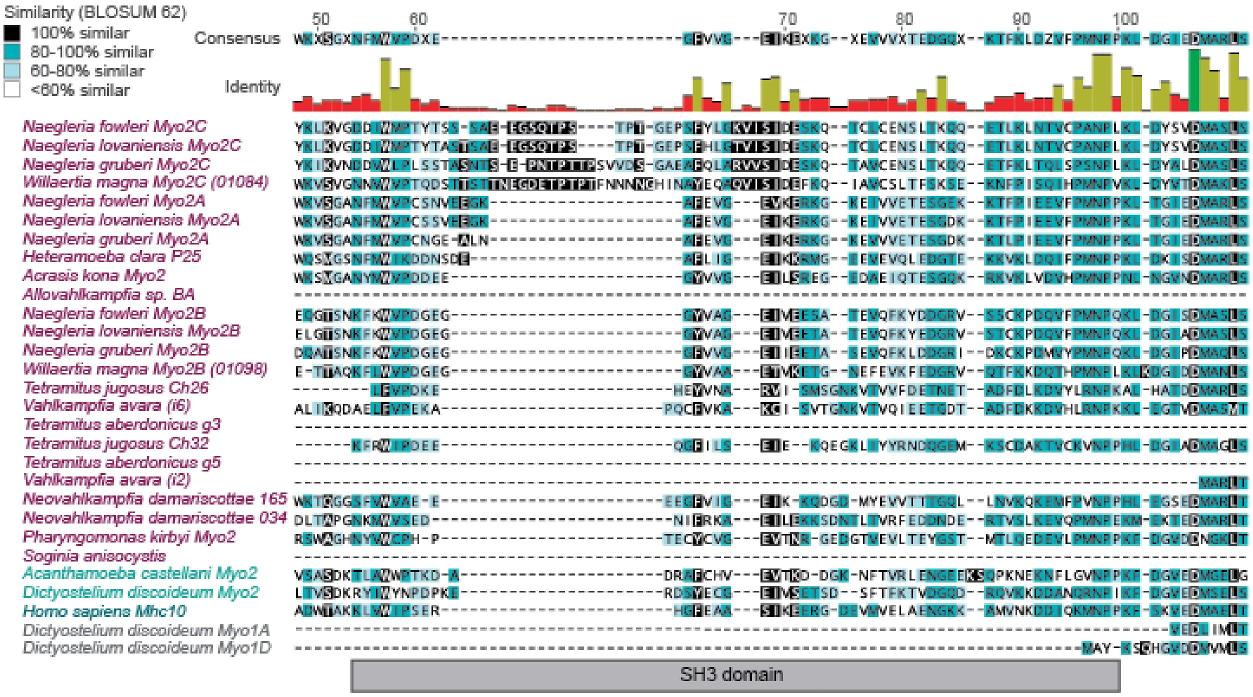

B

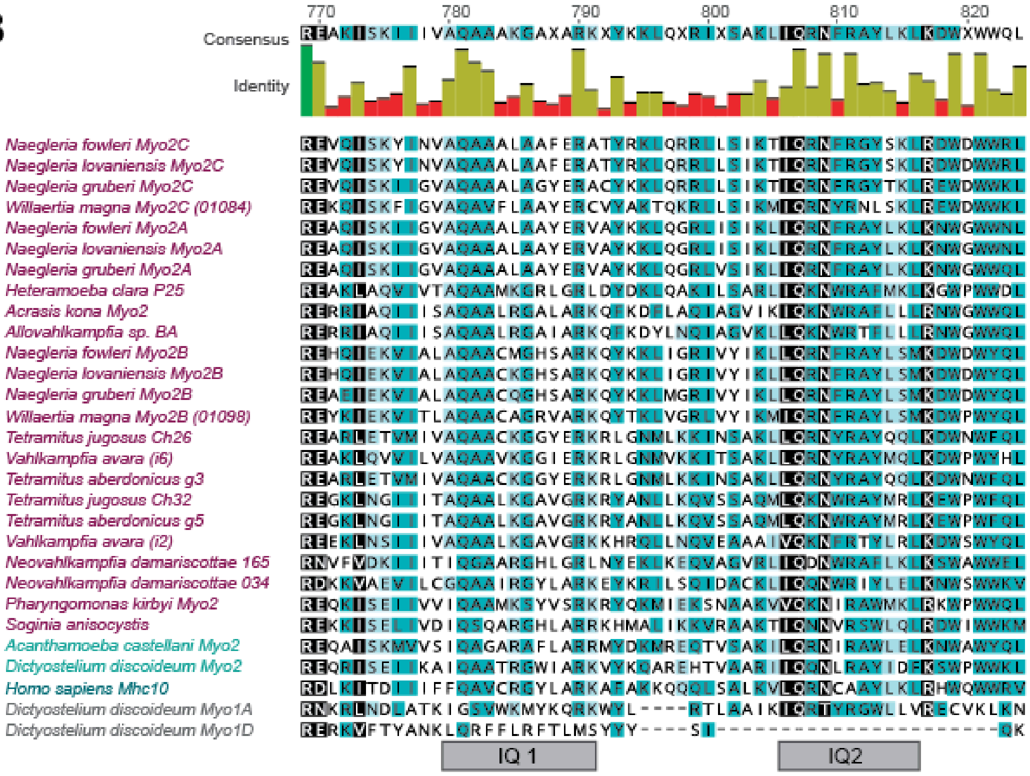

bioRxiv preprint doi: https://doi.org/10.1101/2025.05.16.654244; this version posted May 19, 2025. The copyright holder for this preprint (which was not certified by peer review) is the author/funder, who has granted bioRxiv a license to display the preprint in perpetuity. It is made available under aCC-BY 4.0 International license.

Figure S4: Similarity across regions outside the Myo2 motor domain
The SH3 domain at the N-terminus of Myosin 2 proteins contributes to its kinetic properties, actin affinity, and regulator interactions.[53,54,86] (**A**) A multi-sequence alignment using ClustalO shows heterolobosean sequence similarity of the SH3 domain to the alignment consensus, colored by percent similarity (black = 100% BLOSUM62, teal = 80-100%, light blue = 60-80%, and white = <60%). The Heteroloboseans' SH3 regions had variable scores with less than 80% similarity to the *Dictyostelium* Myo2 reference (average 31.5%, compared to *Acanthamoeba* at 36.4%). (**B**) The regulatory and essential light chains, which are critical to Myosin 2 function, interact with the two IQ motifs in the neck region beyond the motor domain.[57,87] The IQ motifs in this alignment generally follow the IQ motif consensus. By pairwise comparison to the *Dictyostelium* Myosin 2 reference, the first IQ motif averaged 56.5% similar for Heteroloboseans, (vs *Acanthamoeba* 75%), and the second IQ motif was 67.6% similar for Heteroloboseans, (vs *Acanthamoeba* 90.9%).

bioRxiv preprint doi: https://doi.org/10.1101/2025.05.16.654244; this version posted May 19, 2025. The copyright holder for this preprint (which was not certified by peer review) is the author/funder, who has granted bioRxiv a license to display the preprint in perpetuity. It is made available under aCC-BY 4.0 International license.

SUPPLEMENTAL DATASET AND MOVIE LEGENDS

Dataset S1: A spreadsheet with manually selected ROIs and colocalization analyses shown in **Figure 3A** and **Figure S1**. This spreadsheet has four tabs: Flagellate ROI, Amoeba ROI, Flagellate Colocalization, and Amoeba Colocalization. Rows in ROI tabs correspond to paired images of individual cells stained for Myosin 2, actin (green) and tubulin (gray). Each image shows the ROI (yellow box) used for colocalization measurements of both Myo2:actin and tubulin:actin. The Pearson's correlation coefficients (R) were measured for each ROI at 0° and 90° rotations. In the Flagellate and Amoeba Colocalization tabs, the Pearson's correlation coefficients are organized first by antibodies used (Myo2A-C and tubulin), then by degree of ROI rotation (0 and 90). Below these columns are the averages for each ROI's R-value ("Average Pearson's"), SE for all R-values, and the combined averages and SE for all 3 sets of both Myo2:actin and tubulin:actin, indicated by 0 or 90 degrees.

Movie S1: A video showing three example ghosts of *Dictyostelium, Naegleria,* and *Vahlkampfia* contracting upon addition of Mg-ATP. Each time series is labeled, and each series was adjusted by removing time points prior to treatment to show Mg-ATP addition at approximately the same time. All three ghosts are shown at the same scale (Time = minutes:seconds, scale bar = 10 μm).
